# Comparative Study of CO_2_ Capture by Adsorption in Sustainable Date Pits-Derived Porous Activated Carbon and Molecular Sieve

**DOI:** 10.3390/ijerph18168497

**Published:** 2021-08-11

**Authors:** Mohd Danish, Vijay Parthasarthy, Mohammed K. Al Mesfer

**Affiliations:** 1 Chemical Engineering Department, College of Engineering, King Khalid University, Abha 61411, Saudi Arabia; almesfer@kku.edu.sa; 2 Chemical Engineering Department, University of Petroleum and Energy Studies, Dehradun 248001, India; pvijay@ddn.upes.ac.in

**Keywords:** breakthrough, CO_2_ uptake, capacity utilization factor, biomass, mass transfer zone

## Abstract

The rising CO_2_ concentration has prompted the quest of innovative tools to reduce its effect on the environment. A comparative adsorption study using sustainable low-cost date pits-derived activated carbon and molecular sieve has been carried out for CO_2_ separation. The adsorb ents were characterized for surface area and morphological properties. The outcomes of flow rate, temperature and initial adsorbate concentration on adsorption performance were examined. The process effectiveness was investigated by breakthrough time, adsorbate loading, efficiency, utilized bed height, mass transfer zone and utilization factor. The immensely steep adsorption response curves demonstrate acceptable utilization of adsorbent capability under breakthrough condition. The adsorbate loading 73.08 mg/g is achieved with an 0.938 column efficiency for developed porous activated carbon at 298 K. The reduced 1.20 cm length of mass transfer zone with enhanced capacity utilization factor equal 0.97 at 298 K with C_in_ = 5% signifies better adsorption performance for date pits-derived adsorbent. The findings recommend that produced activated carbon is greatly promising to adsorb CO_2_ in fixed bed column under continuous mode.

## 1. Introduction

CO_2_’s increasing volume in the atmosphere is extremely causative to the rise in Earth’s average surface temperature. The utmost overbearing problem is the alarming pace by which the CO_2_ volume in the atmosphere is accelerating [1]. The accessible CO_2_-reducing techniques have the potential to cut CO_2_ emission cost. The many suggested techniques can be adapted to existing plants that rely on non-renewable fuel sources [2,3,4]. An adsorption is considered a prominent technology to separate CO_2_ from emissions through a post-combustion process [5,6]. An adsorbent first adsorbs an adsorbate and, successively, regenerates to release CO_2_ [7]. Technologically achievable porous adsorbents were examined extensively to analyze an adsorption performance [8,9,10,11,12,13]. A number of biomass-derived adsorbents have been transformed into porous carbons for CO_2_ separation by adsorption. The microwave irradiation was applied to develop activated carbon from macadamia shell-derived biomass [14]. Porous fiber was synthesized from biomass waste and reported a good adsorbate loading equal to 1.3 mmol/g [15]. An adsorbent developed by impregnating coal with KOH predicted an adsorbate capacity of 9.1 mmol/g [16].

An olive residue-derived adsorbent was developed by activating the adsorbent with CO_2_ and subsequently heat treating with ammonia [17]. The CO_2_ adsorption loading was established to increase in the presence of nitrogen functionalities predominantly at low pressure. An activated carbon was developed into a porous adsorbent by applying hydrothermal techniques for CO_2_ adsorption [18]. An adsorbate loading equal to 1.45 mmol/g was reached at 0 °C for adsorbent prepared using physical activation by CO_2_. An activated carbon was developed using pyrolysis of white wood for CO_2_ adsorption and the influence of activation techniques was examined [19]. The maximal adsorbate loading of 1.8 mmol/g was accomplished for an adsorbent prepared by impregnation with KOH. Biomass-derived appropriate porous material was developed by impregnation of biomass using KOH [20]. The loading of 5 mmol/g was achieved by utilizing an adsorbent developed by chemical activation of hydrochar with potassium hydroxide under 1 bar pressure. An improved CO_2_ adsorption was reported for activated carbon developed from black locust by KOH induced activation [21] with higher 7.19 mmol/g CO_2_ loading at 0 °C. The wheat flour-derived adsorbent was prepared for CO_2_ separation with enhanced loading of 5.70 mmol/g at 0 °C [22]. The wheat bran was used to synthesize ash and equivalent pellets, and loading of 0.07 mmol/g pronounced for ash pellets prepared at 25 °C [23]. Waste pomegranate peel-derived and KOH-impregnated adsorbent was developed and CO_2_ loading of 1.3 mmol/g was attained [24].

The adsorbent was developed from coffee grounds by combination of ammoxidation and KOH activation and CO_2_ capture capacity was determined by Thermogravimetric analyzer [25]. The maximal CO_2_ capacity of 2.67 mmol/g with a good CO_2_ selectivity over N_2_ of 74.2 was reported at 35 °C. The spent coffee grounds were used to prepare microporous carbon by solid-state K_2_CO_3_ activation for CO_2_ capture [26]. The maximal CO_2_ capacity of 4.54 mmol/g at 25 °C and 1 atm was reported for the activated carbon prepared by activation at 700 °C for 5 hr. Surface modified activated carbon was developed from corncob-based hydrochar for CO_2_ adsorption [27] and KOH impregnated activated carbon showed the maximal adsorption uptake of 4.5 mmol/g at 1 bar and 15 °C.

The potassium hydroxide impregnated adsorbent was prepared from nut-based biomass and 5.5 mmol/g CO_2_ loading was attained for a peanut shell at 900 °C temperature [28]. It was acclaimed that the existence of extra oxygen-containing groups increased the adsorbent loading for olive stone-derived activated carbon [29]. A considerably good loading of 101.7 mg CO_2_/g was shown at 30 °C for porous carbon developed from biomass [30]. The rapeseed/walnut blend was carbonized and, after that, subjected to high temperature to synthesize porous carbon-based adsorbent [31]. Similarly, biomass based on walnut exploited to produce activated carbon for preparing the cartridge [32]. The high-performance adsorbents were made using various shells, and the structure was examined by FTIR and SEM [33]. The potassium hydroxide is recognized as a favorable activating agent to prepare carbon-based adsorbent from walnut shells [34]. Researchers [35] synthesized porous adsorbent from walnut shell, and investigated the separation ability of C_6_H_6_ by treating with ZnCl_2_/H_3_PO_4_. Walnut shell-based porous activated carbon was developed and 1.6 mmol/g capacity of adsorption was calculated at 298 K [36].

The raw date pits-derived porous adsorbent was prepared by impregnation with KOH and a capacity of 123.1 mg/g was accomplished [37]. An adsorbent was made from date pits applying steam activation with good porosity and tailored O_2_ surface groups [38]. A far-reaching review on exploiting data pits as an adsorbent for various applications was performed [39]. An activated carbon derived from date pits was developed for CO_2_ adsorption and 141.14 mg/g CO_2_ loading was stated [40]. An improved CO_2_ loading equal to 3.5 mmol/g was achieved for an adsorbent prepared from date seeds at an activation temperature of 900 °C [41]. A porous carbon-based adsorbent was developed using date sheets and a higher 6.39 mmol/g CO_2_ loading was reached at 1 bar and 0 °C [42]. The adsorption effectiveness of carbon-based adsorbent from date seed for CO_2_ adsorption was assessed [43] and established that existence of H_2_O may not substantially impact the adsorption performance.

In Saudi Arabia, the date pits-based biomass material is economically obtainable in huge quantity. The preparation of date pits-derived carbon-based adsorbent specially for CO_2_ separation by adsorption using continuous mode has not been examined fully. The originality of the present study is to develop a porous adsorbent from date pits for CO_2_ adsorption and equating the adsorption performance with molecular sieve 3Å. The performance of CO_2_ capture was determined considering feed rate, temperature and initial adsorbate volume as operating variables.

## 2. Material and Methods

### 2.1. Materials

The commercially available MS-3Å with an average diameter of 1.60 mm was procured from Sigma-Aldrich (Steinheim, Germany). The date stones-based biomasses were purchased from the regional market. These collected biomasses were rinsed with distilled H_2_O a number of times to remove dust particles and the sample was dried overnight at 150 °C. The size reduction of date pits was carried out using Grinder. The milled biomass particles were then passed through 4 mm screen (BS series). The particles were stored in air-proof containers for further use.

### 2.2. Experimental Unit

**Figure 1:**
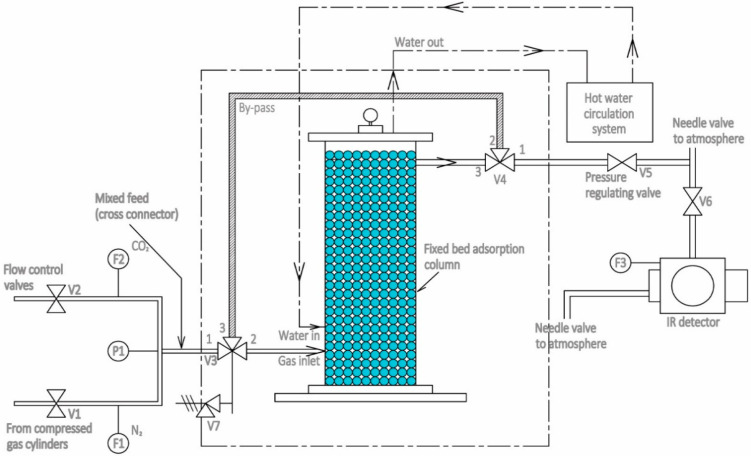
Fixed bed column.

Setup used for the experimental work is shown in Figure 1. The 23 cm is the operative length of column packed by porous adsorbent. The flow rates of both the gasses are controlled by mass flow controllers. The feed made of CO_2_/N_2_ passes into the column from bottom side. The mass flow controllers F1 and F2 measure and control the flow rates of N_2_ and CO_2_, respectively. The hot water circulator equipped with control console is used to achieve the desired set point temperature in the column. The required flow to IR sensor for estimating the CO_2_ concentration in outgoing gas from column is measured by other flow controller F3. Various thermocouples positioned inside the column are used to determine temperature variation during adsorption–desorption process.

### 2.3. Procedure

The mixture of CO_2_/N_2_ is allowed to enter the fixed column as per the configuration shown in Figure 1. IR sensor measures the CO_2_ concentration at adsorbent bed outlet at desired regular interval of times. The uncertainty values for the measurements using F1, F2, and F3 in addition to that for thermocouple are shown in Table 1. After the completion of adsorption process, desorption of adsorbent bed was carried out by continuously flowing the N_2_ for a sufficient period of time. The complete desorption of the bed was ensured by measuring the bed outlet CO_2_ concentration (C_exit_), where C_exit_ = 0% was measured up to 3 places after the decimal point. The accuracy of F1, F2 and F3 were determined in addition to that for thermocouple. An accuracy analysis for all the sensors is depicted as seen in Table 1. The accuracy of the temperature sensor was measured equal to ±0.15 K. An accuracy with associated with IR sensor for flow controller F3 was determined as ±0.01 lpm. An accuracy of ±0.04 lpm and ±0.02 lpm corresponded to flow controllers F1 and F2, respectively.

### 2.4. Physical Activation

**Figure 2:**
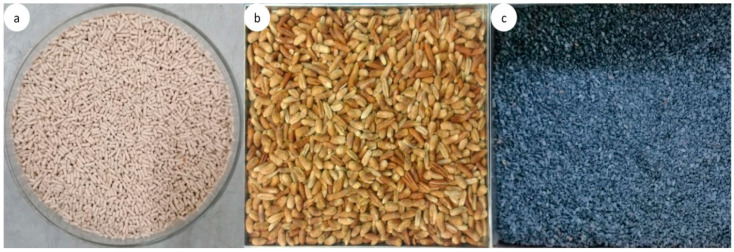
(**a**) MS 3Å, and (**b**) oven-dried date pits and (**c**) produced porous activated carbon AC-SY.

The MS 3Å used for comparative purpose has been shown in Figure 2a. An activated carbon (AC-SY) was developed from date pits-based biomass by physical activation using a multi-zone tubular furnace (Model: OTF-1200X) (MTI Corporation, Richmond, CA, USA). The furnace consists of three zones that can be used to control dissimilar temperatures at the same time span. A known sieved date stone weight was placed in the inner side the tube of furnace and afterwards, the temperature was raised at 10 °C/min with a N_2_ flow equal to 150 mL/min to accomplish the needed 600 °C. The N_2_ was constantly maintained for an extra 2 h for carbonization. After that, the N_2_ flow was closed and CO_2_ flow allows constantly for 2 h at 600 °C to develop the desired carbon (AC-SY). The collected date stones and synthesized porous activated carbons are depicted in Figure 2b,c There was considerable reduction in adsorbent size after the activation. The produced AC-DS of average size 1.35 mm were selected for the experimental study.

## 3. Results

### 3.1. Morphological and Surface Characteristics

The produced activated carbons (AC-SY) were characterized using NovaWin (Quantachrome: Boynton Beach, FL, USA) analyzer for surface with 573 K outgas temperature. The analysis period was 88.3 min and N_2_ was supplied for analysis purpose. The surface characterization results for AC-SY and MS-3Å are presented (Table 2). The surface area (single point) of 149.11 m^2^/g was acquired for synthesized adsorbent. The 0.45 cm^3^/g and 0.02 cm^3^/g pore volumes were observed for AC-SY and MS 3Å, respectively. The pore radius of activated carbon is larger compared to that of molecular sieve. Largely, the adsorbents are vastly porous materials. The sorption takes place mostly either at definite sites within the particles or on the pore walls.

**Figure 3:**
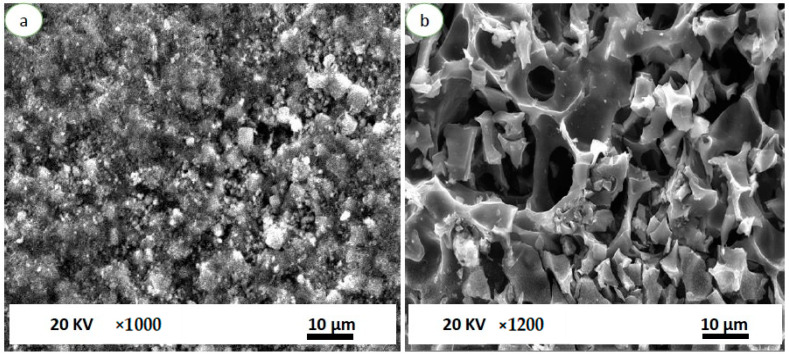
SEM images of (**a**) MS-3Å, and (**b**) AC-SY.

The surface morphology of MS-3Å and AC-SY was analyzed by the SEM (Model: Quanta 250-FEI; Thermo Fisher Scientific: Brno, Czech Republic). The structural images have been depicted in Figure 3a for MS 3Å and Figure 3b for AC-SY. The various pores can be visualized and the pores nearly spread wholly above the developed porous structure, as presented in Figure 3b with 1200× level and Figure 3a with 1000× level for MS-3Å. The very regular and high-density pore structures are especially effective for CO_2_ separation.

**Figure 4:**
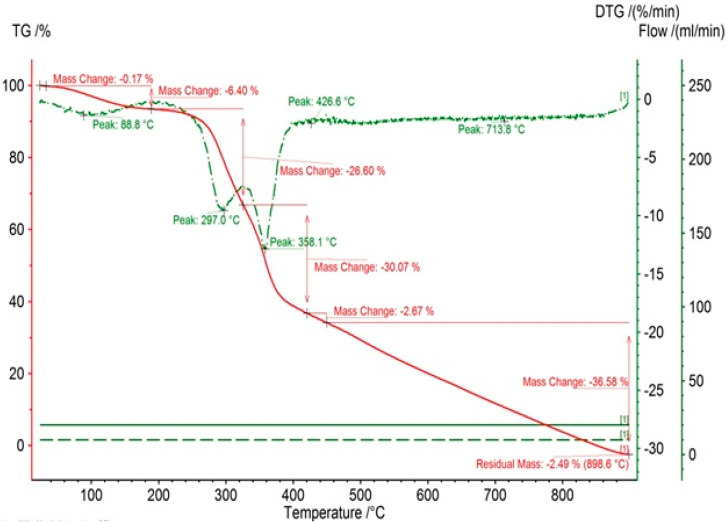
TGA response curve for biomass presenting change of mass and DGT.

**Figure 5:**
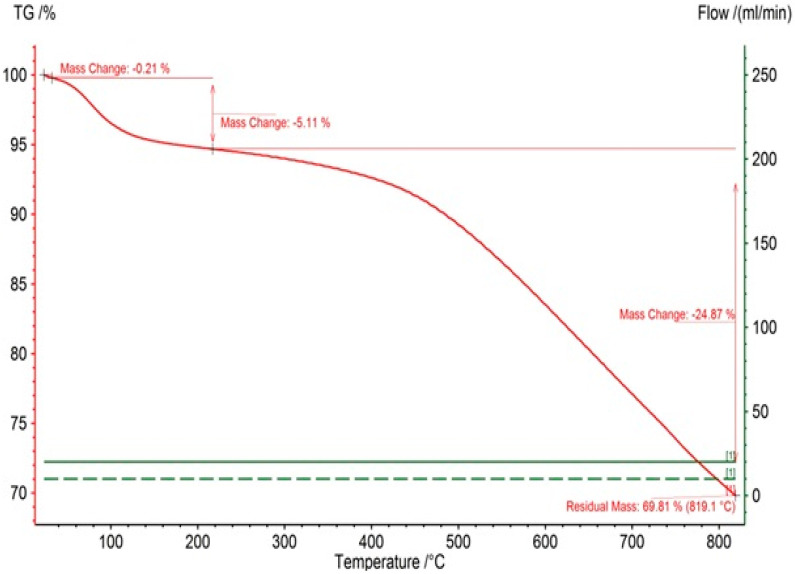
TGA response curve of produced activated carbon.

The thermal stability of the developed structure was examined by Thermogravimetric analyzer (TG209 FI Libra). The temperature reliance on TG (%) and DTG (% change/min) has been depicted in Figure 4 for biomass. A biomass sample equal to 18.43 mg was placed in the analyzer and temperature was raised to 900 °C with temperature incremental rate of 20 °C/min. N_2_ purge was fixed at 20 mL/min and flow rate of protective gas adjusted equal to 10 mL/min. The dependence of weight loss on temperature is determined by proximate analysis. The temperature equivalent to which the loss in weight is more apparent is typically specified by the derivative. It is a useful method to define the optimum temperature and time conditions. The variation of TG (%) on temperature for porous-activated carbon has been presented in Figure 5. A mass change of 0.21% and 5.1% have been observed at a temperature of less than 50 °C and 250 °C, respectively. The residual mass amounts to 69.81% at a temperature of 819.1 °C.

**Figure 6:**
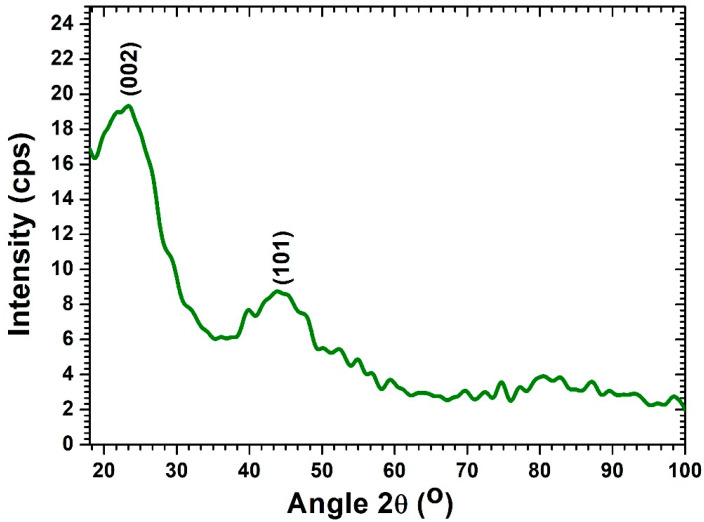
XRD of date pits derived AC-SY.

The synthesized activated carbon (AC-DS) was examined using XRD (Model: X’Pert Pro) (Malvern Panalytical, Malvern, U.K.). The scan range 2θ = 0–100° was chosen to analyze X-ray patterns as depicted in Figure 6. An unstructured form of biomass-based adsorbent is attributed to the presence of broad peaks. These are the preferred characteristics of produced carbon for CO_2_ capture by adsorption. The peaks of diffraction at 2θ = 23.96° and 45.34° are envisioned from produced adsorbent which are analogous to the amorphous carbon peaks. Nearly the same diffraction peaks for developed activated carbon-based adsorbents were also observed as reported in the literature [26,44]. The broad (002) diffraction peak (2θ = 15–30°) can be credited to the amorphous structure of prepared carbon adsorbent. The broad and weak (101) diffraction peak (2θ = 40–50°) is owing to the *a* axis of graphite structure. The absence of sharp peaks in the produced activated carbon signifies that the carbon structure is mainly amorphous which is beneficial for well-defined adsorbent.

### 3.2. Breakthrough Analysis

**Figure 7:**
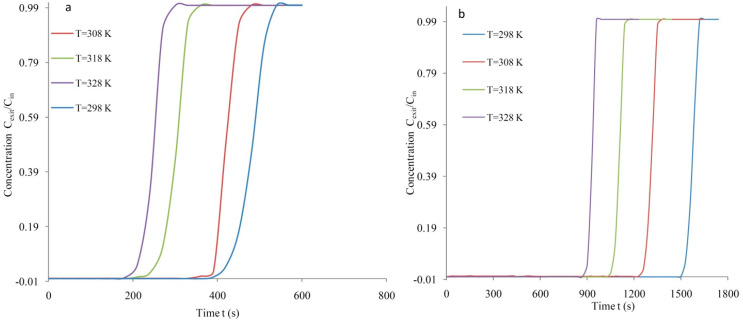
Adsorption response at various temperatures at F = 4 lpm, C_o_ = 5%: (**a**) MS-3Å, and (**b**) AC-SY.

The feed flows were measured and regulated by provided flow controllers with control console. The data were collected at 4 lpm feed flow with an adsorbate concentration of 5% (vol.%). The operating pressure was fixed at 1.25 bars. The adsorption response curves produced at different temperature for MS-3Å (Mass, m_ad_: 230 g) have been depicted in Figure 7a. The breakthrough span of 417 was determined with a saturation period of 513 s at 298 K. The breakthrough period declined to 388 s with increased temperature at 308 K. The breakthrough period lessens to 250 s for MS-3Å with an increased temperature at 318 K. The saturation and breakthrough periods of 210 s and 283 s have been determined at 328 K. The lengthy breakthrough or exhaustion spans are always well-thought-out essential for the improved CO_2_ capture. At large, the prolonged breakthrough time at reduced temperatures recommends increased CO_2_ loading.

The breakthrough curves for AC-SY (Mass, m_ad_: 150 gm) generated under various temperature have been depicted (Figure 7b). The breakthrough period relies significantly on the temperature at which the adsorption takes place. Generally, the time it takes to reach 5% of the maximal concentration is called breakthrough time. The prolonged saturation and breakthrough periods relate to decreased temperature at 298 K. The extended breakthrough periods of 1524 s were attained at a temperature of 298 K. The breakthrough period declined to 1265 s with raised 308 K temperature. The breakthrough and saturation condition arrived prematurely corresponding to 1059 s and 1138 s with 318 K temperature. The highest studied temperature of 328 K was accredited to a reduced breakthrough period of 900 s. Prolonged exhaustion/or breakthrough time spans have been comprehended for AC-SY comparative to MS 3Å. The adsorption response profiles produced for AC-SY were comparatively steeper compared to that obtained for MS-3Å.

The better performance of the bed for CO_2_ adsorption was thoroughly established by the steepness of breakthrough curve as evident from Figure 7b. The breakthrough curves generated for AC-SY were relatively steep compared to the breakthrough curves obtained for MS 3Å. The utilization of required CO_2_ loading conforming to breakthrough state is favored for profitable adsorption of adsorbate CO_2_ from feed. The abruptness of adsorption response profiles attributed to the narrowness of mass transfer zone length. A lesser MTZ zone characterizes to faster uptake of CO_2_. The variations in molecular weight result in the occurrence of adsorption. Additionally, several molecules held more firmly than others upon the adsorbent surface owing to polarity. In numerous instances, the adsorbate held firmly and sufficiently to permit inclusive capture of CO_2_ from feed with very small or no sorption of non-absorbable gas. Indeed, steepness of adsorption response profiles depicted in Figure 7b specifies comparable utilization of CO_2_ loading.

**Figure 8:**
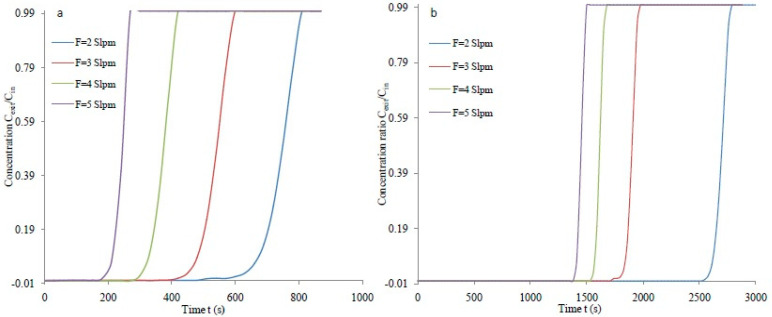
Flow rate dependence of adsorption profiles at C_in_ = 5%, T = 298 K, (**a**) MS-3Å and (**b**) AC-SY.

Figure 8a depicts the dependence of feed flow on breakthrough pattern for MS-3Å. The feed flows ranging from 2 to 5 lpm have been selected at 1.25 bars with 298 K temperature. The 2 lpm flow contributed to a breakthrough period of 650 s that is significantly lower than the period reported for AC-SY. The breakthrough period was perceived to reduce to 460 s with an increased feed flow to 3 lpm. The increased feed rate of 4 lpm attributed to reduced exhaustion and breakthrough spans of 311 s and 415 s with 298 K and C_in_ = 5%, respectively. The breakthrough time of 200 s was determined at highest studied 5 lpm feed rate with T = 298 K. Consequently, the breakthrough and exhaustion periods decrease suggestively with increased feed rate consisting of one of the components as an adsorbate.

The feed flow dependence on adsorption response profiles generated for AC-SY was presented in Figure 8b. The exhaustion and breakthrough times depend extraordinarily on flow rate of feed. The extended breakpoint and saturation times of 2603 s and 2769 s were observed at a flow of 2 lpm. The time equivalent to C_exit_/C_in_ = 0.05 C_in_ was seen to reduce from 2603 s to 1833 s with raised from 2 to 3 lpm at 298 K. Further, the breakthrough period declined to 1564 s with augmented 4 lpm. The minimal breakthrough period of 1401 s was achieved for activated carbon with 5 lpm feed flow. It was seen clearly that the prolonged breakthrough and exhaustion times are observed for activated carbon compared with MS 3Å at any predetermined feed rate.

The adsorption response profiles formed under various feed rate for activated carbon are very steep (Figure 8b). Correspondingly, an enhanced bed capacity utilization was characterized by narrow mass transfer zone observed for adsorption response profiles. The sharpness of the profiles is useful for the economical separation of CO_2_ from feed mixture. The C_exit_ increases rapidly, equivalent to the profiles ending at the state where the adsorbent is considered as unproductive and this happens when the breakthrough point is attained.

**Figure 9:**
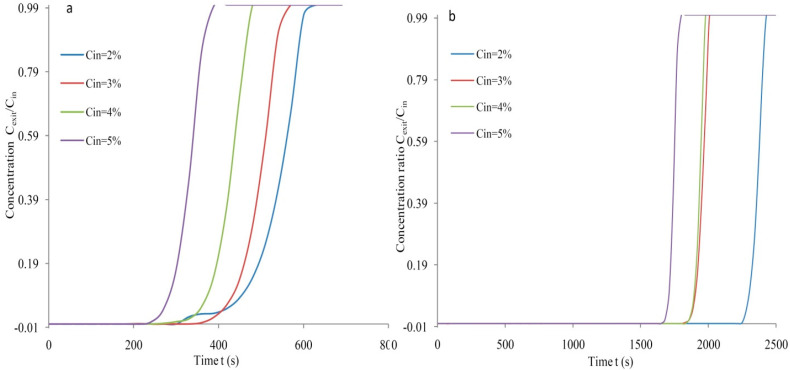
Adsorption response at various CO_2_ level at F = 4 lpm, T = 298 K: (**a**) MS-3Å, and (**b**) AC-SY.

The reliance of C_in_ on the breakthrough profiles for MS-3Å has been depicted in Figure 9a under the same operating conditions of feed flow (F = 4 lpm) and temperature (T = 298 K). The different initial adsorbate concentration ranging from 2 to 5% were controlled with operating 1.25 bars of pressure. The adsorbate concentration C_in_ = 2% in feed at 298 K attributed to a breakthrough period of 420 s at 4 lpm. The exhaustion and breakthrough periods were identified to reduce to 543 s and 413 s with an increased level of initial adsorbable gas in feed at 3% level. The breakthrough period further reduced to 360 s with an increased C_o_ to 4% with 298 K and 4 lpm. The exhaustion period further diminished to 380 s on increasing the CO_2_ level at 5% under the fixed operating condition of feed flow. The prolonged breakthrough and exhaustion times are always required for increased CO_2_ uptake.

The initial adsorbate concentration dependence on breakthrough profiles at F = 4 lpm with 298 K are depicted in Figure 9b for AC-SY. The breakthrough period is perceived to lessen with increased adsorbate concentration in feed. The minimal adsorbate concentration of 2% contributes to exhaustion period of 2422 s. The breakthrough period reduced to 1884 s on increasing the adsorbate concentration level equal to 3% (vol.%). The 4% adsorbate concentration has revealed early arrival of exhaustion period of 1976 s. Utmost 5% adsorbate concentration in the feed forecast the breakthrough time of 1695 s and established that the augmented initial CO_2_ amount documented to the early appearance of exhaustion/breakthrough periods. It was obviously observed that exhaustion and breakthrough spans are longer for biomass-based activated carbon compared to that determined for MS 3Å. The breakthrough span is proportional to the CO_2_ loading and varies reciprocally with feed concentration.

### 3.3. Adsorption Capacity and Column Efficiency

The CO_2_ loading is estimated by using material balance. Utilizing the adsorption response profiles, stoichiometric period (*t_s_*) equivalent to the total capacity may be calculated [45] as:

(1) $$t_{s}=\int_{0}^{\infty }\left(1-\frac{C_{exit}}{C_{in}}\right)dt\;$$

The CO_2_ loading *q_s_* (mg/g) is determined [46] as:

(2) $$q_{s}=\frac{F\;t_{s}C_{in\;}}{m_{ad}}$$

where *m_ad_*: mass of sorbent, *F*: feed rate, *C*: adsorbate concentration at time t, *C_in_*: initial CO_2_ level in feed.

Up to the breakthrough time *t_b_*, the time equal to the usable capacity can be calculated as:

(3) $$t_{b}=\int_{0}^{t}\left(1-\frac{C_{exit}}{C_{in}}\right)dt\;$$

The corresponding column capacity until the breakthrough point can be calculated as:

(4) $$q_{b}=\frac{F\;t_{b}C_{in}}{m_{ad}}$$

The faction of total bed capacity which is efficiently utilized (η):

(5) $$\eta =\frac{q_{b}}{q_{s}}=\frac{\;\;\;\;\int_{0}^{t}\left(1-\frac{C_{exit}}{C_{in}}\right)dt\;\;\;\;\;\;\;\;\;}{\int_{0}^{\infty }\left(1-\frac{C_{exit}}{C_{in}}\right)dt}$$

The length of adsorption column used up to the breakpoint Equation (6):

(6) $$L_{b}=\frac{t_{b}}{t_{s}}L\;$$

where *L*: total bed length, an unusable bed length can be calculated as:

(7) $$L_{ub}=\left(1-\frac{t_{b}}{t_{s}}\right)\;L\;$$

The adsorption performance for MS 3Å and AC-SY determined at different temperatures has been summarized as depicted in Table 3. The CO_2_ uptake varies substantially with temperature for both types of adsorbents and alters negatively with temperatures. The maximal CO_2_ uptake (q) of 63.18 mg/g was attained for AC-SY equated to a capacity of 13.83 mg/g for MS-3Å at 298 K with 4 lpm. The maximal 328 K temperature characterized by minimal uptake and CO_2_ uptake of 33.09 mg/g was determined for AC-SY. All the CO_2_ capture system predicts a reduction in the uptake with an increased temperature. The prepared activated carbon contributed to higher effective column efficiency (η) as evident from the Table 3. Additionally, the column efficiency (η) based on column capacity reported for synthesized carbon is considerably higher with a highest value 0.944 at 298 K. The fraction of total capacity is effectively used is 0.845 for MS-3Å at 298 K. Significantly, higher efficiency of AC-SY indicates its feasibility for economical CO_2_ capture. The effective column length (L) for the adsorption is fixed at 23 cm. Additionally, the bed length used up to the breakthrough point is 19.44 cm at 298 K for MS 3Å. The maximal column length of 21.71 cm was used in case of AC-SY which is higher than reported for MS-3Å. Industrially, the essential cycle period and feed flow decides the size of adsorber. Applying a bed length of less than or equal to 12 cm is occasionally suggested to reduce adsorber height and drop in pressure; however, an empty column needs more regeneration energy and does not offer inclusive separation. It can be suggested that produced activated carbon performs very well and suitable for CO_2_ separation from CO_2_/N_2_ feed.

The adsorption performance for MS-3Å and AC-SY determined at various feed flows has been summarized and depicted in Table 4. The CO_2_ uptake varies positively and substantially with feed flow for both types of adsorbents. The maximal 73.08 mg/g CO_2_ loading was determined for AC-SY at 298 K with 5 lpm. The lowest studied feed flow of 2 lpm contributed to a minimal capacity of adsorption and CO_2_ uptake of 8.54 mg/g was determined for MS-3Å at the 298 K and C_in_ = 5%. The adsorption performance of developed activated carbon is reasonably higher at any fixed feed flow. The produced activated carbon contributed to higher effective column efficiency as evident from the Table 4. Additionally, the efficiency based on column efficiency reported for synthesized carbon is considerably higher with a highest value of 0.940 at 2 lpm or 3 lpm with 298 K and C_in_ = 5%.

The highest fraction of total capacity effectively used is 0.890 for MS-3Å with a feed flow of 2 lpm under the same condition of temperature and initial absorbable gas level. In general, it was seen that effective column efficiency reduces marginally with increased feed flow from 2 to 5 lpm. Significantly, higher efficiency of AC-SY dictates its suitability for economical CO_2_ separation from CO_2_/N_2_ mixture. The usable bed height up to the breakthrough reduces with increased feed flows for either type of adsorbent. Additionally, the bed length utilized up to the breakthrough point is 20.47 cm at 2 lpm with 298 K for MS-3Å. The maximal bed length of 21.62 cm was utilized in case of activated carbon which is higher than reported for MS-3Å. It can be predicted that effective column efficiency and bed length utilized up to the breakthrough point varies negatively with augmented feed flow rates. The unutilized bed length of MS-3Å is more in comparison to activated carbon. It can be suggested that developed activated carbon performs very well and suitable for CO_2_ separation from CO_2_/N_2_ feed.

The adsorption performance for MS 3Å and AC-SY evaluated as a function of initial adsorbable gas concentrations has been outlined in Table 5. The CO_2_ uptake varies largely and positively varies with initial concentration of adsorbable gas in feed. The lowest and highest CO_2_ uptakes of 7.94 mg/g and 12.31 mg/g were attained for MS-3Å at 2% and 5% CO_2_ level, respectively. The maximal CO_2_ uptake of 70.13 mg/g was determined for AC-SY compared to a lower value reported for MS-3Å at C_in_ = 5% with 298 K and 4 lpm feed flow rate. The lowest C_in_ = 2% contributed to a minimal capacity of adsorption and CO_2_ uptake of 37.93 mg/g was evaluated for AC-SY. The CO_2_ uptake varies proportionally with the initial adsorbable gas level. The adsorption performance of developed activated carbon is reasonably higher at any fixed initial adsorbable gas level in feed.

The prepared activated carbon contributed to higher effective column efficiency as evident from the Table 5. Additionally, the efficiency (η) determined for synthesized carbon is higher and highest η of 0.949 was evaluated at C_in_ = 4% CO_2_ level in feed with 4 lpm and 298 K. The highest fraction of total capacity which is effectively used stands at 0.760 for MS-3Å at 3% CO_2_ level with 4 lpm. Notably, the higher efficiency of AC-SY indicates its applicability for economical CO_2_ capture. Additionally, longer bed length L_b_ was utilized for AC-SY up to the breakthrough point compared to the bed height utilized for MS-3Å for an effective bed length of 23 cm. The maximal bed length of 21.84 cm was utilized in case of AC-SY which is higher than the value obtained for MS-3Å. The developed activated carbon performs satisfactorily in terms of various characteristics parameters. Table 6 summarized the adsorption capacity reported under different operating conditions for date-based activated carbon and molecular sieve 3Å.

### 3.4. Zone of Mass Transfer

**Figure 10:**
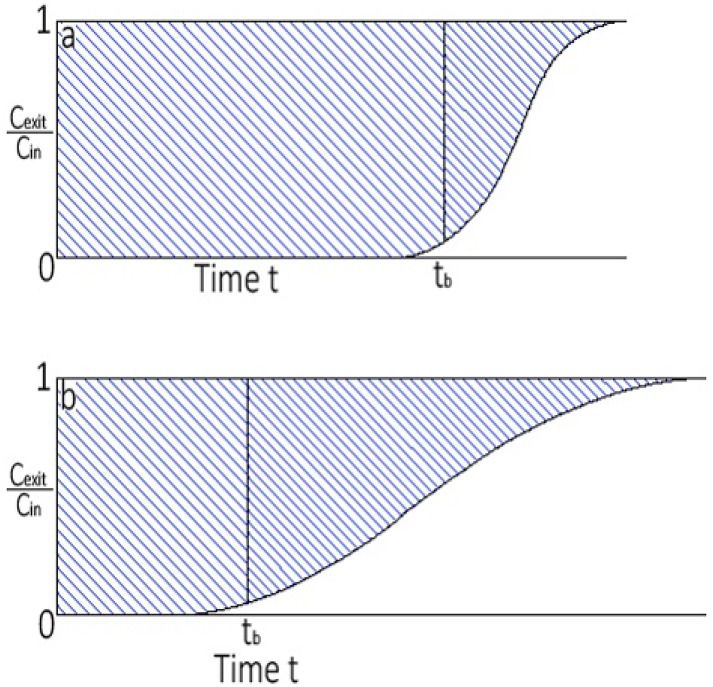
(**a**) Breakthrough profile and narrow *MTZ*, and (**b**) breakthrough profile and wide *MTZ*.

An adsorbate amount in solid phase and fluid phase differs as a function of time and the location inside the column. At earliest, the basic CO_2_ movement occurs in the vicinity of the bed input and feed comes in contact with fresh porous sorbent. The CO_2_ concentration in the feed relinquishes drastically with position effectively to zero before the end point of column is reached provided that porous adsorbent has no CO_2_ at the start of adsorption. The section of the adsorption bed in which adsorbate is adsorbed or portion of the bed in which adsorbate amount varies mostly is widely known as zone of mass transfer (L_MTZ_). The concentration limits are normally assumed as C_exit_/C_in_ = 5 to 95% (vol.%). A lean MTZ attributes to the efficient utilization of the adsorbent resulting in reduced regeneration cost. MTZ usually moves from the input to the output side suggesting that the adsorbent adjacent to inlet position attained the state of saturation for adsorbate, subsequently the MTZ shifts towards the exit side of the column. Figure 10a,b demonstrates breakthrough curves with narrow and wide mass transfer zones. The *L_MTZ_* [47] was calculated assuming steady pattern or CO_2_ adsorption utilizing the Equation (8):

(8) $$L_{\mathrm{MTZ}}=\frac{\;2\;L\left(t_{s}-t_{b\;}\right)}{t_{s}+t_{b\;}}$$

where *t_s_*: exhaustion time; *t_b_*: breakthrough time and L: effective column height. The area above the adsorption response profiles to the breakthrough period (*t_b_*) denotes the actual amount adsorbed. The profile very much extended in the situation of L_MTZ_ is approximately equal to column length. The mass transfer zone is characterized by insignificant width supposing that there is not at all resistance to mass transfer. Under such conditions, the adsorption response profiles possibly remain a perpendicular line between C_exit_/C_in_ = 1.0 and 0 when an adsorbent is saturated entirely. The porous sorbent is wholly saturated between the bed inlet and the start of the *L_MTZ_* under the breakthrough condition. The adsorbent under the *MTZ* goes from nearly saturated to almost no adsorbate, and for a rough average this sorbent material possibly believed to be nearly 50% saturated. For the assumed adsorption response profiles as uniform, the f may be determined:

(9) $$f=1-\;\frac{\;0.5\;L_{MTZ}}{L}$$

The characteristics parameters of the CO_2_ adsorption are determined for MS-3Å as presented in Table 7. The breakthrough period reduces with raised column temperatures. The factor f differs marginally with column temperature. Reduced zone of mass transfer results in an increased utilization factor. The shortest L_MTZ_ equivalent to 2.28 cm results in good utilization factor of 0.950 at 298 K with F = 3 lpm and C_in_ = 5%. The breakthrough and exhaustion spans reduce with increased mixture flow containing adsorbate ranging 2 to 5 lpm. The feed rate variations contribute considerably to L_MTZ_ and f as evident from the findings. The L_MTZ_ of 2.28 cm was realized at feed flow rate of 3 lpm with a utilization factor to 0.950 at 298 K and C_in_ = 5%. The declined f equal to 0.855 is determined at the maximal feed rate of 5 lpm under constant conditions. The breakthrough period also differed with initial adsorbate volume. The f estimated at various adsorbate volume did change remarkably under constant operating conditions of temperature and feed flow. Largely, the smaller L_MTZ_ indicates good utilization factor. The L_MTZ_ equivalent to 2.23 cm suggests the satisfactory usage of bed capacity. The utmost f = 0.950 determined at 298 K and C_in_ = 5%. An improved utilization factor is continually considered necessary for reasonable adsorption of adsorbate gas.

The characteristic parameters of the CO_2_ adsorption calculated for AC-SY have been depicted (Table 8). The breakthrough period lessens with augmented temperatures in the examined temperature range. The shortest L_MTZ_ of 1.20 cm for effective bed length of 23 cm was realized at 298 K leading to increased utilization factor of 0.974 cm with F = 4 lpm and C_in_ = 5%. The factor f changes adversely with augmented temperature and maximal value was reported at a temperature of 298 K. Smaller L_MTZ_ is always desired for efficient separation of CO_2_ from feed mixture resulting in increased utilization factor. The breakthrough and exhaustion times reduce with raised feed flows in the range of 2 to 5 lpm. The feed rate variations contribute marginally to L_MTZ_ and f as evident from the findings. The nearly the same L_MTZ_ and f were determined at the feed flow of 2 and 3 lpm with fixed T = 298 and C_in_ = 5% and the CO_2_ utilization factor of 0.696 was determined at the same feed flows. The exhaustion and breakthrough period also vary with initial adsorbate volume. The factor f estimated at various initial adsorbate volume do vary notably under constant operating conditions. The increased utilization factor equal to 0.974 was attained with L_MTZ_ = 1.20 cm at adsorbate volume of 4% at 298 K with 4 lpm feed flow. Largely, high utilization factor is characterized by smaller mass transfer zone.

**Figure 11:**
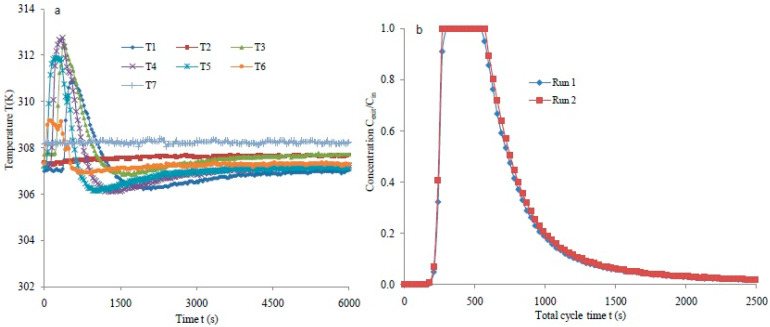
(**a**) Temperature curves for MS-3Å at F= 4 slpm and C_in_= 5%, and (**b**) repeatability assessment utilizing MS-3Å. at F = 4 slpm, C_in_ = 5% and T = 328 K.

The temperature profiles measured by different thermocouples are presented in Figure 11a. The data were collected for MS-3Å. The temperature of the hot-water circulator was preset at 308 K using PID controller. The thermocouples are placed at different locations inside the bed. In general, a temperature increase of about 10 (383 K) to 50 °C (323 K) may possibly occurs on treating vapors with merely 1% adsorbable component. The temperature rise is restricted by heat loss for a bed of small diameter, but a big adsorber will function approximately adiabatically. Furthermore, increased adsorbate volume, the heat liberated during the adsorption leads to increased temperatures as verified by measured temperatures. Repeatability analysis (Figure 11b) was performed in case of MS-3Å to determine the re-applicability of the measurement. The experimental data were measured at 4 lpm feed flow, C_in_ = 5%, with 328 K. The IR sensor provided for measuring the column exit CO_2_ concentration provides accurate results for maximal CO_2_ concentration of 5% (vol.%) in the feed. The R^2^ = 0.994 confirmed a good correlation between the obtained experimental data. The standard deviation σ = ±0.012 (vol.%) was observed as measured by IR detector. The σ value describes outstanding conformism between the measured quantities. A study focused on the deactivation and regeneration of amine-modified adsorbent for CO_2_ adsorption was conducted [48] and it was suggested that such amine-containing adsorbent materials may be recycled for use many time without any loss of performance. A CO_2_ adsorption and adsorbent stability study over primary, secondary and tertiary monoamine-grafted pore-grafted mesoporous MCM-41 silicas was carried out [49] and it was suggested that only primary amines suffered significant loss in CO_2_ adsorption capacity. It was recommended that repeatability was satisfactory to trust upon the obtained data.

## 4. Conclusions

The dependence of temperature, feed flow and initial CO_2_ level on breakthrough and exhaustion periods is very significant and these periods vary considerably with the operating parameters. It was clearly demonstrated that exhaustion and breakthrough spans are longer for produced carbon from date stones compared to that for MS-3Å under different set of operating conditions. The adsorption profiles produced for AC-SY were hugely steep, validating an outstanding utilization of capacity under breakthrough condition. The best 13.83 mg/g CO_2_ loading was achieved at 4 lpm with T = 298 K and C_in_ = 5% for MS-3Å. The MS-3Å also contributed to an effective column efficiency of 0.89 with used bed length of 19.44 cm. The maximal 73.08 mg/g CO_2_ loading was determined at 5 lpm with T = 298 and C_in_ = 5% for produced adsorbent derived from biomass. The date pits-derived porous carbon accomplished good column efficiency of 0.949 with satisfactory usable bed height equal to L_b_ = 21.84 cm. The MS-3Å was also characterized by L_MTZ_ = 2.28 cm and f value of 0.950 at 3 lpm with T = 298 K and C_in_ = 5%. The developed carbon found to be better with reduced L_MTZ_ = 1.20 cm and enhanced f of 0.974 with 298 K, C_in_ = 4% and F = 4 lpm. It was clearly observed that synthesized carbon from economical date pits-derived biomass performs better compared with the MS-3Å. It is recommended that exploiting the biomass based on date pits for the synthesis of porous adsorbents is frugally convincing to adsorb CO_2_ by adsorption.

## Figures and Tables

**Table 1:** Determination of accuracy of measuring sensors.

Variable (u)	Sensor Type	Uncertainty (δu)
N_2_ flow rate (slpm)	F1	±0.04
CO_2_ flow rate (slpm)	F2	±0.02
IR flow rate (slpm)	F3	±0.01
Temperature (K)	Thermocouple type-K	±0.15

**Table 2:** Surface characterizations of MS-3Å and AC-SY.

Adsorbent Characteristics	MS-3Å	AC-SY
Single point surface (m^2^/g)	26.42	149.11
Multiple point surface area	26.87	463.80
Langmuir surface area (m^2^/g)	46.21	1444.0
DFT Pore volume (cm^3^/g)	0.02	0.46
DFT Pore radius (Å)	8.44	9.23

**Table 3:** Adsorption characterizations at different temperatures (F = 4 lpm, C_in_ = 5%).

MS-3Å				AC-SY		
T(K)	q (mg/g)	η (fr.)	L_b_ (cm)	q (mg/g)	η (fr.)	L_b_ (cm)
298	13.83	0.845	19.44	63.18	0.944	21.71
308	11.64	0.834	19.18	50.60	0.938	21.57
318	8.24	0.746	17.16	44.45	0.931	21.41
328	6.74	0.742	17.07	33.09	0.939	21.60

**Table 4:** Adsorption performances at various feed rates (T = 298 K, C_in_ = 5%).

MS-3Å				AC-SY		
F (lpm)	q (mg/g)	η (fr.)	L_b_ (cm)	q (mg/g)	η (fr.)	L_b_ (cm)
2	10.33	0.890	20.47	54.30	0.940	21.62
3	11.52	0.776	17.80	57.55	0.940	21.62
4	10.73	0.749	17.23	65.43	0.939	21.60
5	8.54	0.746	17.16	73.08	0.938	21.57

**Table 5:** Dependence of adsorption performances on initial adsorbate levels (T = 298 K, F = 4 lpm).

MS-3Å				AC-SY		
C_in_ (vol.%)	q (mg/g)	η (fr.)	L_b_ (fr.)	q (mg/g)	η (fr.)	L_b_ (cm)
2	7.94	0.702	16.15	37.93	0.941	21.65
3	10.93	0.760	17.48	46.95	0.940	19.74
4	12.12	0.758	17.43	61.82	0.949	21.83
5	12.31	0.711	16.35	70.13	0.948	21.84

**Table 6:** CO_2_ adsorption capacity of various adsorbents.

Adsorbent	Temperature (°C)	Pressure (bar)	Adsorption Capacity (mg/g)	References
Date pits-AC	40	0.14	118.8	[37]
Date pits-AC	20	1	154.0	[41]
Date sheet-AC	0	1	281.6	[42]
Date sheet-AC	25	1	193.6	[42]
Date sheet-AC	25	40	968.0	[42]
Date pits-AC	25	1.3	73.08	[Current work]
Molecular sieve 3Å	25	1.3	13.93	[Current work]

**Table 7:** Summary of characteristic CO_2_ capture parameters of MS-3Å.

T (K)	F (slpm)	C_in_ (vol.%)	t_b_ (s)	t_s_ (s)	L_MTZ_ (cm)	f
298	4	5	417	513	4.75	0.897
308	4	5	388	459	3.86	0.916
318	4	5	250	335	6.67	0.855
328	4	5	210	283	6.81	0.852
298	2	5	650	803	4.84	0.895
298	3	5	460	593	2.28	0.950
298	5	5	200	268	6.68	0.855
298	4	2	420	598	8.04	0.825
298	4	3	413	543	6.26	0.864
298	4	4	360	475	5.53	0.880

**Table 8:** Characteristic parameters of CO_2_ adsorption for produced porous carbon.

T (K)	Q (slpm)	C_in_ (vol.%)	t_b_ (s)	t_s_ (s)	L_MTZ_ (cm)	f
298	4	5	1524	1615	1.33	0.971
308	4	5	1265	1348	1.46	0.968
318	4	5	1059	1138	1.65	0.964
328	4	5	900	958	1.44	0.969
298	2	5	2603	2769	1.42	0.969
298	3	5	1833	1951	1.43	0.969
298	5	5	1401	1494	1.48	0.968
298	4	2	2280	2422	1.39	0.970
298	4	3	1884	2005	1.43	0.969
298	4	4	1875	1975	1.20	0.974

## Data Availability

Not applicable.
